# Adaptive Laboratory Evolution Reveals the Selenium Efflux Process To Improve Selenium Tolerance Mediated by the Membrane Sulfite Pump in Saccharomyces cerevisiae

**DOI:** 10.1128/spectrum.01326-23

**Published:** 2023-04-26

**Authors:** Ao Gong, Wenyue Liu, Yelong Lin, Laili Huang, Zhixiong Xie

**Affiliations:** a Hubei Key Laboratory of Cell Homeostasis, Wuhan University, Wuhan, China; b College of Life Sciences, Wuhan University, Wuhan, China; University of Melbourne

**Keywords:** adaptive laboratory evolution, selenium tolerance, selenium efflux, Se-enriched yeast, selenium, yeasts

## Abstract

Selenium (Se) is a micronutrient in most eukaryotes, and Se-enriched yeast is the most common selenium supplement. However, selenium metabolism and transport in yeast have remained unclear, greatly hindering the application of this element. To explore the latent selenium transport and metabolism mechanisms, we performed adaptive laboratory evolution under the selective pressure of sodium selenite and successfully obtained selenium-tolerant yeast strains. Mutations in the sulfite transporter gene *ssu1* and its transcription factor gene *fzf1* were found to be responsible for the tolerance generated in the evolved strains, and the selenium efflux process mediated by *ssu1* was identified in this study. Moreover, we found that selenite is a competitive substrate for sulfite during the efflux process mediated by *ssu1*, and the expression of *ssu1* is induced by selenite rather than sulfite. Based on the deletion of *ssu1*, we increased the intracellular selenomethionine content in Se-enriched yeast. This work confirms the existence of the selenium efflux process, and our findings may benefit the optimization of Se-enriched yeast production in the future.

**IMPORTANCE** Selenium is an essential micronutrient for mammals, and its deficiency severely threatens human health. Yeast is the model organism for studying the biological role of selenium, and Se-enriched yeast is the most popular selenium supplement to solve Se deficiency. The cognition of selenium accumulation in yeast always focuses on the reduction process. Little is known about selenium transport, especially selenium efflux, which may play a crucial part in selenium metabolism. The significance of our research is in determining the selenium efflux process in Saccharomyces cerevisiae, which will greatly enhance our knowledge of selenium tolerance and transport, facilitating the production of Se-enriched yeast. Moreover, our research further advances the understanding of the relationship between selenium and sulfur in transport.

## INTRODUCTION

Selenium (Se) is an essential trace element in human nutrition that widely participates in the formation of selenoproteins and enzymes required for antioxidation, inflammation reduction, thyroid hormone production, DNA synthesis, fertility, and reproduction (1, 2). Due to rapid proliferation and efficient assimilation of many minerals from the environment, yeast cells are commonly used for the production of food and fodder for humans and livestock in the biotechnology industry, especially for the production of selenium supplements. Se-enriched Saccharomyces cerevisiae is one of the most popular sources of selenium supplements (3). During the process of enrichment, yeasts are cultured with inorganic Se sources, selenite (SeO_3_^2−^) or selenate (SeO_4_^2−^), to create low-molecular-weight and less toxic organic Se compounds, while selenomethionine (SeMet) is the dominant and bioavailable form in humans and animals (4). Considering the extensive application of Se-enriched yeasts, focusing on the mechanism of selenium accumulation and its metabolism in yeast cells is worthwhile.

Generally, selenium metabolism in yeast principally refers to other elements adjacent to the periodic table, especially sulfur (S). Selenium has high chemical/physical similarity to S; for this reason, Se compounds follow the same metabolic routes as those of S compounds (5). Selenium reduction depends primarily on glutathione (GSH), and selenium assimilation is conducted by sulfur metabolism pathways (4, 6). A few previous studies have noted that the process of intracellular selenium accumulation occurs mainly through the active transport of other ions inside yeast cells. The known selenium transport systems include the sulfate permeases SUL1 and SUL2 for selenate (7) and monocarboxylic conveyer JEN1 and low-affinity (PHO87, PHO90, and PHO91)/high-affinity (PHO84 and PHO89) phosphate transporters for selenite (8, 9). However, until now, there have been no reports related to the selenium efflux process, which may play a crucial part in selenium metabolism and the production of Se-enriched yeast. Consequently, it is still necessary to explore the unknown selenium transport and metabolism mechanisms.

Adaptive laboratory evolution (ALE) is a powerful method to improve strain tolerance against artificial stress and expand the current understanding of molecular mechanisms that confer stress tolerance (10). Under the influence of the proper selective pressure, a microbial strain is propagated continually for hundreds of generations until the desired phenotypes emerge because of the accumulated mutations in the evolution process (11). Combined with whole-genome resequencing, ALE helps investigate the links between the genotype and the phenotype of evolved strains. The genetic basis or potential metabolic mechanism related to tolerance against selective pressure can be easily determined (12). Previously, ALE was frequently used to explore the mechanisms of tolerance to diverse substances in S. cerevisiae, such as glycerol (13), urea (14), weak acids (15), and salinity (16). In most cases, the accumulated mutations during ALE can lead to an elevated metabolism capacity in the cell against selective pressure (17, 18). In other cases, enhanced transporters, or efflux pumps, that are responsible for the generated tolerance can be identified by researchers (19, 20). Finally, based on the findings of ALE, the yield of the corresponding target products can be improved (19).

**FIG 1:**
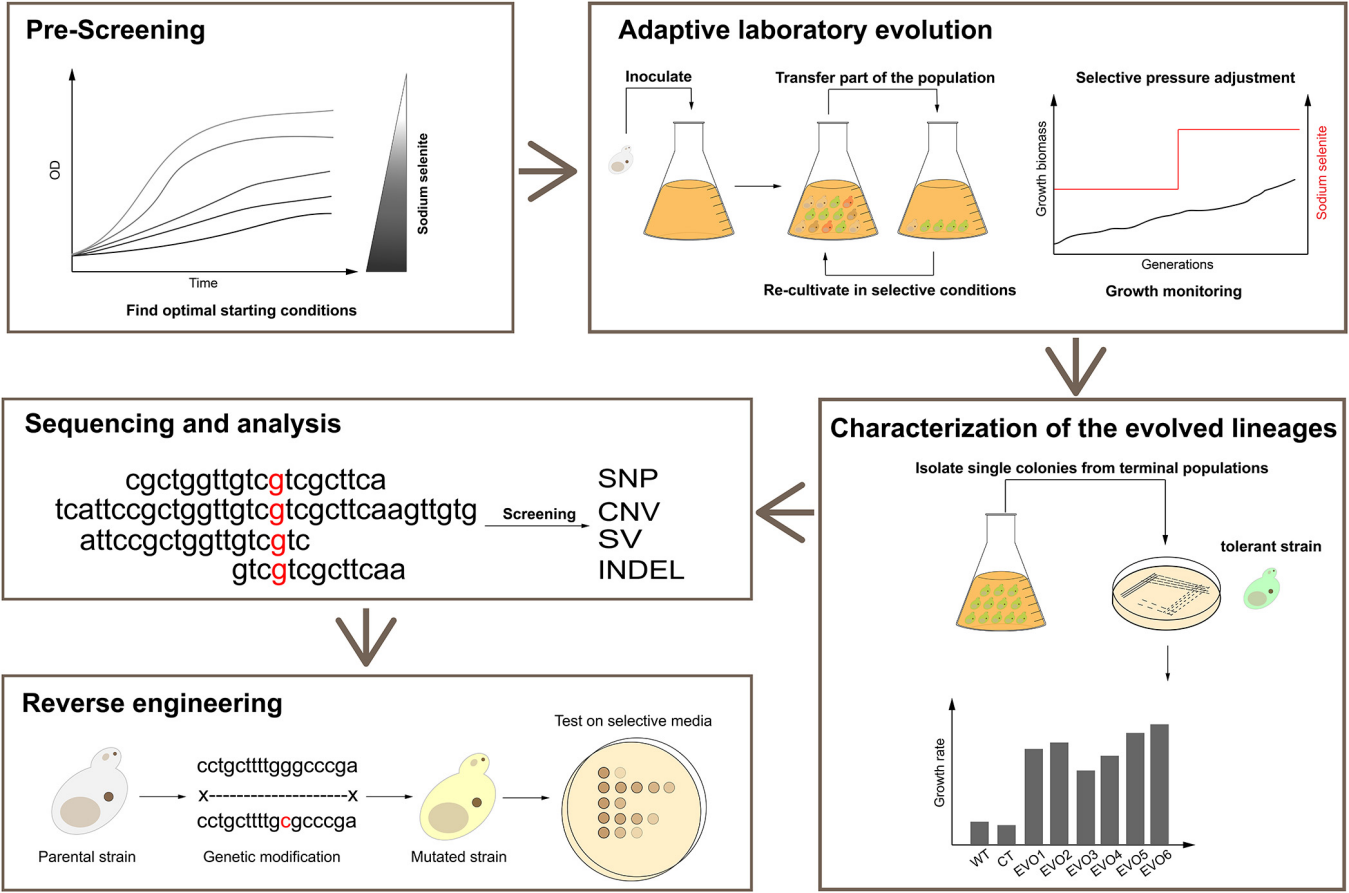
Scheme of the experimental procedures to investigate selenium tolerance in S. cerevisiae. For prescreening, the growth inhibition of parental strains was measured in the presence of different concentrations of sodium selenite, and the optimal starting conditions were found and applied for subsequent serial cultivations. For adaptive laboratory evolution, strains were serially transferred into fresh YPD medium with the addition of increasing concentrations of sodium selenite for 100 days. For the characterization of the evolved yeast lineages, six clones were isolated from Ev1 to Ev6, and their selenium tolerance was characterized and evaluated. For sequencing and analysis, whole-genome sequencing of six single clones from E1 to E6 and one clone from the starting group was performed, and potentially functional mutations generated in the evolved strains were screened and identified. For reverse engineering, the determined mutations were introduced into the wild-type strain, and their contribution to the tolerance phenotype was tested.

Inspired by the above-mentioned ALE cases, we employed this method to increase the selenium tolerance of S. cerevisiae to explore the unknown selenium transport and metabolism mechanisms. The workflow used here is summarized in Fig. 1, from the initial determination of the optimal concentration of selenite (prescreening) to the validation of selected mutations (reverse engineering). We found that mutations in the sulfite efflux pump gene *ssu1* and its transcription factor gene *fzf1* are responsible for increased selenium tolerance. Finally, we confirmed the selenium efflux process mediated by *ssu1*, which leads to decreased intracellular selenium accumulation and consequently confers hyper-selenium tolerance to the evolved strains. Overall, our work extends the understanding of selenium metabolism and transport and provides a foundation for the optimization of relevant industrial production.

## RESULTS AND DISCUSSION

### ALE conferred selenium adaptation and resistance to evolved strains.

To determine the suitable selenite concentration before the initialization of the evolution experiment, the growth of S. cerevisiae BY4742 during serial passage with different concentrations of sodium selenite was measured. We found that 50 μM sodium selenite is exactly the starting point to allow the stable reproduction of yeast (data not shown). Three control groups and six evolved groups were set to perform serial transfer for 100 days, and their growth was persistently monitored (see Fig. S1 in the supplemental material).

**FIG 2:**
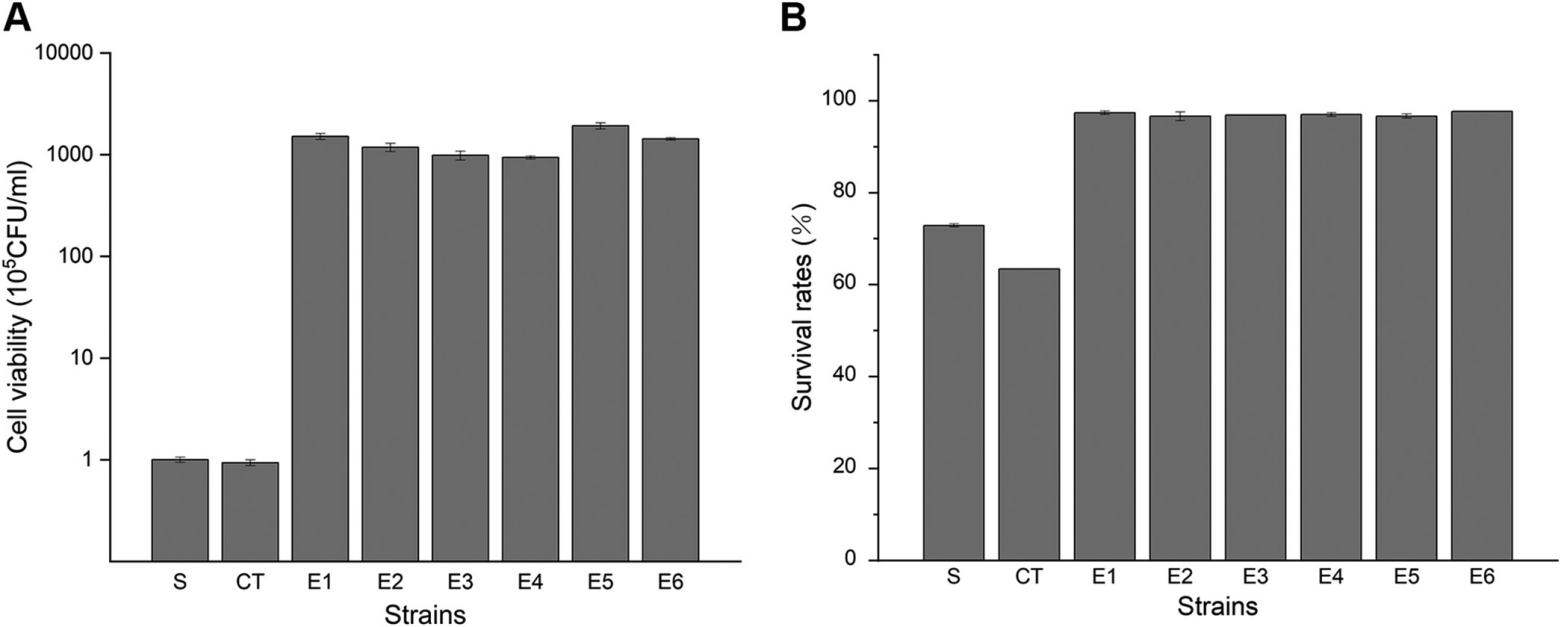
Selenium adaptation of the unevolved and evolved strains. The starting strain is denoted S, the control strain (after serial transfer without sodium selenite) is denoted CT, and the six evolved strains are denoted E1 to E6. (A) Viability of various strains after selenium treatment. Cell samples from all tested strains were harvested after culture in YPD medium with 100 μM Na_2_SeO_3_ for 16 h, diluted, and plated. After incubation at 30°C for 3 days, the number of colonies on the plates was counted. (B) Survival rates of various strains after selenium treatment. Yeast cells were sampled during serial transfer in selenium-containing YPD medium, stained with propidium iodide, and analyzed by flow cytometry. The proportion of unstained cells was regarded as alive. The error bars represent the means and ranges from three independent experiments.

Six strains from the final evolved groups were isolated (denoted E1, E2, E3, E4, E5, and E6), and their selenium adaptation was evaluated by the cell viability and survival rate after selenium treatment. All six evolved strains maintained considerable cell proliferation abilities after incubation with sodium selenite, indicating that they still had high cell viabilities, while the unevolved starting strain (S) and the control strain (CT) showed only very limited cell viability (Fig. 2A). Moreover, after evolving under selenium-selective pressure for 100 days, E1 to E6 could effectively resist continuous selenite stress, and more than 95% of the cells survived, while many cells died in the unevolved S and CT strains (Fig. 2B). Hence, the evolved strains greatly improved their selenium adaptation after long-term evolution from the aspects of cell viability and survival rates.

**FIG 3:**
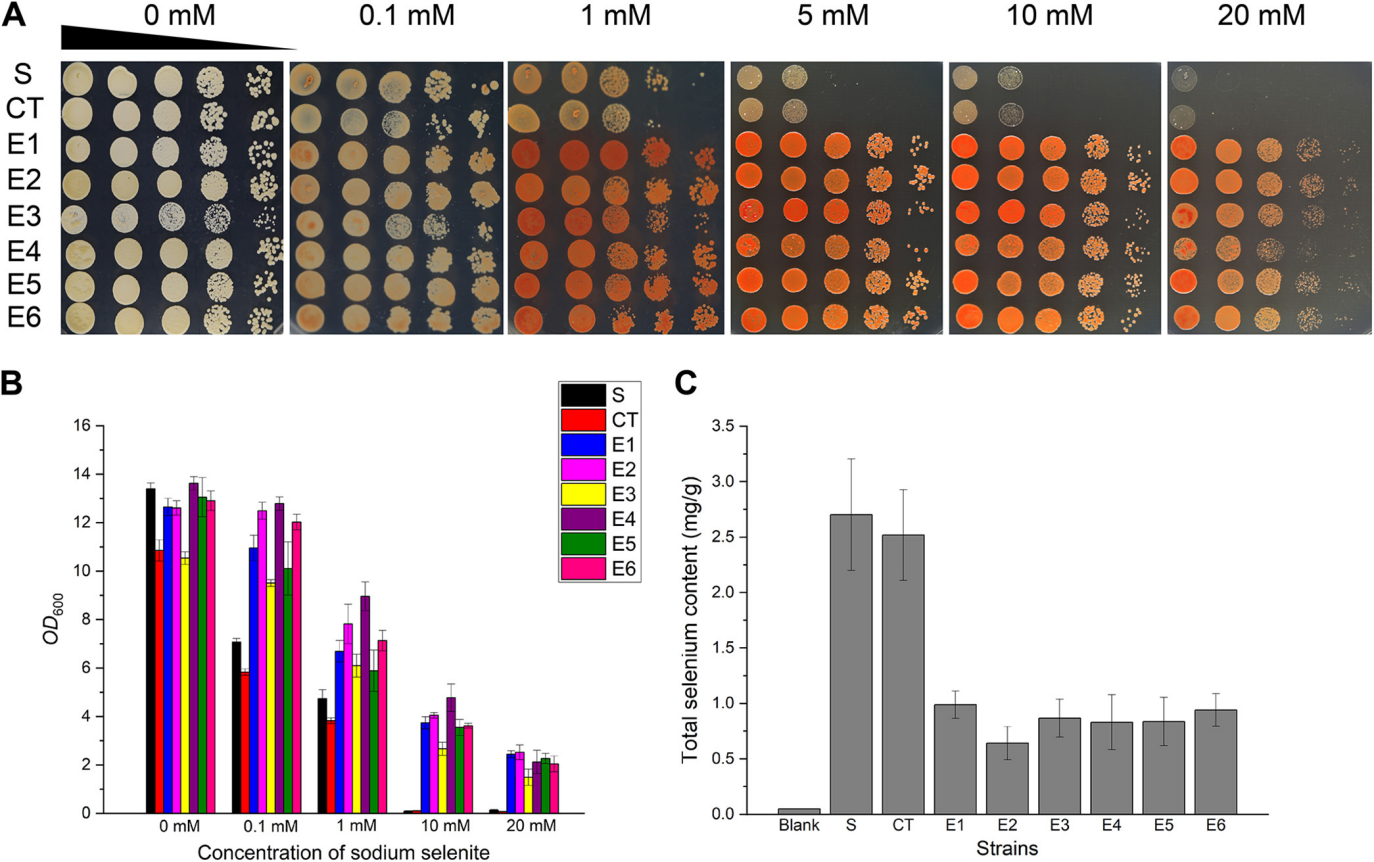
Selenium tolerance and intracellular selenium content in evolved strains. The starting strain is denoted S, the control strain (after serial transfer without sodium selenite) is denoted CT, and the six evolved strains are denoted E1 to E6. (A) Growth assays on agar plates. After incubation in liquid YPD medium overnight, cells were sampled and spotted onto 1.5% YPD agar plates supplemented with increasing concentrations of Na_2_SeO_3_. Pictures were taken 2 days later. (B) Growth assays in liquid medium. All strains were grown at 30°C overnight and then subcultured into fresh YPD liquid medium with increasing concentrations of sodium selenite for 16 h. The final OD_600_ of each strain was measured and recorded. (C) Intracellular selenium content of the evolved strains. After growth in YPD medium with 100 μM Na_2_SeO_3_, yeast cells were sampled and digested, and the selenium content was then analyzed using ICP-MS. The error bars represent the means and ranges from three independent experiments.

Next, we evaluated the selenium tolerance of evolved and unevolved strains in liquid medium and agar plates. With the same trend as the one described above, the evolved strains showed vigorous growth, even when exposed to 20 mM sodium selenite pressure, whether in liquid medium or on agar plates, while the unevolved S and CT strains could not tolerate 10 mM sodium selenite pressure (Fig. 3A and B). The inconspicuous growth defect of CT may come from degeneration during long-term passage in nutritious yeast extract-peptone-dextrose (YPD) medium without selective pressure. Therefore, all six evolved strains successfully generated strong adaptation and tolerance to sodium selenite after laboratory evolution.

To further investigate the possible mechanisms of selenium tolerance in the evolved strains, we measured the intracellular selenium contents of all tested strains. Compared to the unevolved and intolerant S and CT strains, selenium-tolerant strains E1 to E6 had distinctly lower levels of intracellular selenium (Fig. 3C). The lower intracellular selenium content means less selenium-induced toxicity in yeast cells, so the decrease in the intracellular selenium content in E1 to E6 may be the biochemical basis of their tolerant phenotypes. Given that some previous ALE studies of tolerance involved functions of efflux pumps or transporters (20–22), we reasonably speculated that the reduction in the intracellular selenium content in E1 to E6 may be related to an improved selenium efflux capacity. Thus, we performed whole-genome sequencing of S and E1 to E6 to identify potential mutations and verify this hypothesis.

### Mutated *ssu1* and *fzf1* were screened by sequencing analysis.

**FIG 4:**
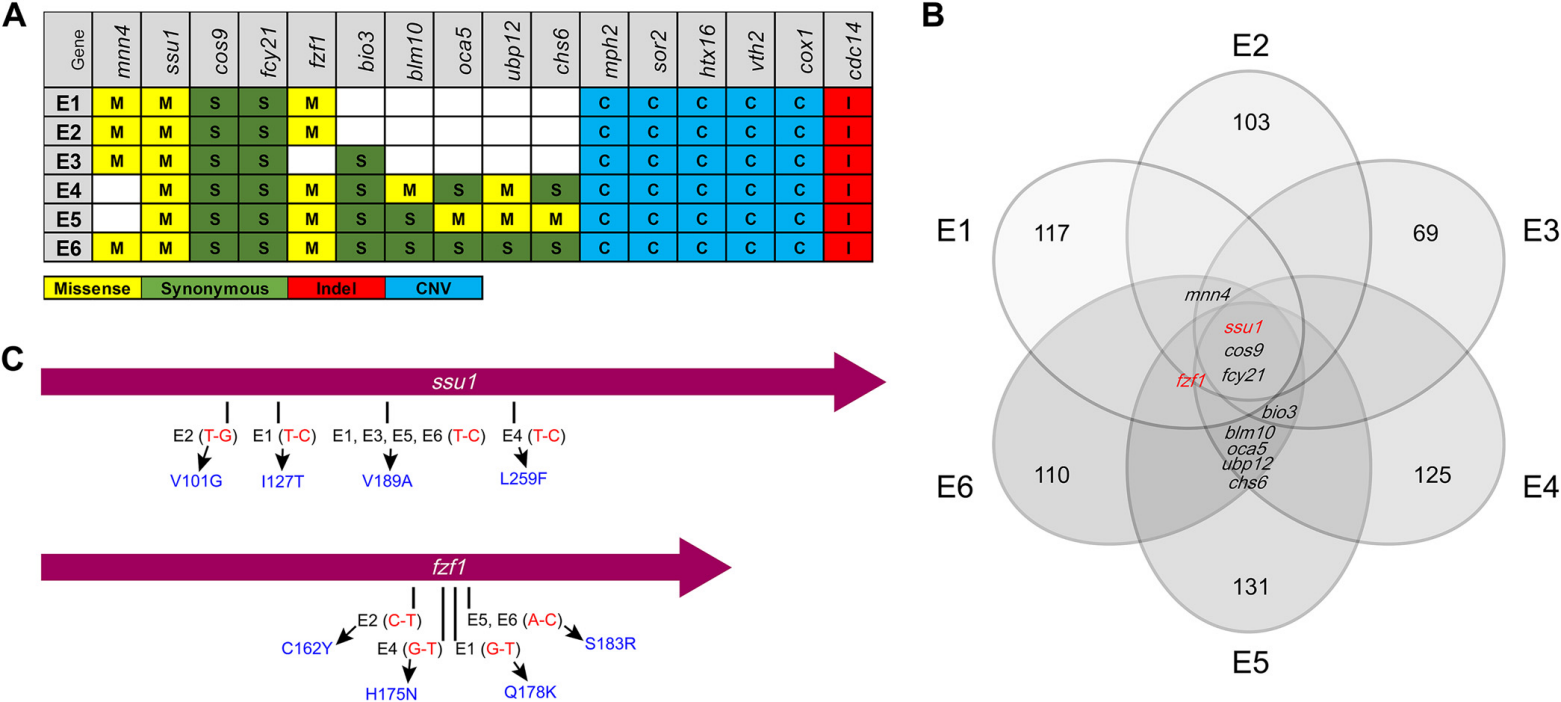
Summary of the mutated genes identified in evolution experiments. (A) Table of gene mutations present in more than three clones from each independently evolved population. (B) Venn diagram of genes with mutations that occurred and accumulated within E1 to E6. Each circle indicates the number of mutated genes found in the clone. *ssu1*, *cos9*, and *fcy21* were found in all six evolved groups simultaneously, and *fzf1* was found in five evolved groups simultaneously except for E3. *mnn4* and *bio3* were found in four evolved groups, while *blm10*, *oca5*, *ubp12*, and *chs6* were found only in E4, E5, and E6. (C) Mutated positions of E1 to E6 in sequence diagrams of *ssu1* and *fzf1*. The length of *ssu1* is 1,377 bp, encoding a 458-aa membrane protein, and the length of *fzf1* is 900 bp, encoding a 299-aa transcription factor. Red represents the base substitution, and blue represents the corresponding amino acid substitution.

Six evolved strains (E1, E2, E3, E4, E5, and E6) and the S strain were analyzed by whole-genome sequencing, and potential mutations related to the selenium-tolerant phenotype were identified. An average genome coverage of 130× was utilized to screen mutations, and the mutations possibly related to the evolution of E1 to E6 are shown in Fig. 4A and B. To identify potential functional mutations and genes, we determined the most frequently mutated genes (*ssu1*, *cos9*, *fcy21*, *fzf1*, *mnn4*, and *bio3*) that simultaneously occurred in more than 4 parallel groups for further functional verification. Notably, *ssu1* is the only gene that occurred with nonsynonymous mutations in all six parallel evolved groups in concert, and missense mutations in *fzf1* existed in five evolved groups (Fig. 4C). Thus, the unusual patterns of mutations of *ssu1* and *fzf1* indicated their paramount effect on selenium tolerance during ALE.

*ssu1* encodes a plasma membrane protein conferring sulfite tolerance to S. cerevisiae (23). Ssu1p is required for efficient sulfite efflux and can directly decrease intracellular sulfite accumulation (24). *fzf1* encodes a transcription factor that is involved in sulfite metabolism (25). Fzf1p is composed of five zinc fingers (ZFs) and plays a positive role in the transcription of *ssu1* (26). Given the tight regulatory relationship between *ssu1* and *fzf1* and that they were both mutated in most evolved groups within several positions coincidentally, these results suggested that *ssu1* and *fzf1* are likely to perform homogeneous functions in selenium metabolism combined with the chemical similarity between selenium and sulfur (27–29). Therefore, we investigated the latent functions of *ssu1* and *fzf1* in the selenium tolerance of yeasts and then confirmed that both *ssu1* and *fzf1* indeed play vital roles, while the other four most frequently mutated genes (*fcy21*, *cos9*, *mnn4*, and *bio3*) screened by sequencing analysis were found to be irrelevant for selenium tolerance (Fig. S2).

### *ssu1* and *fzf1* are involved in selenium tolerance.

**FIG 5:**
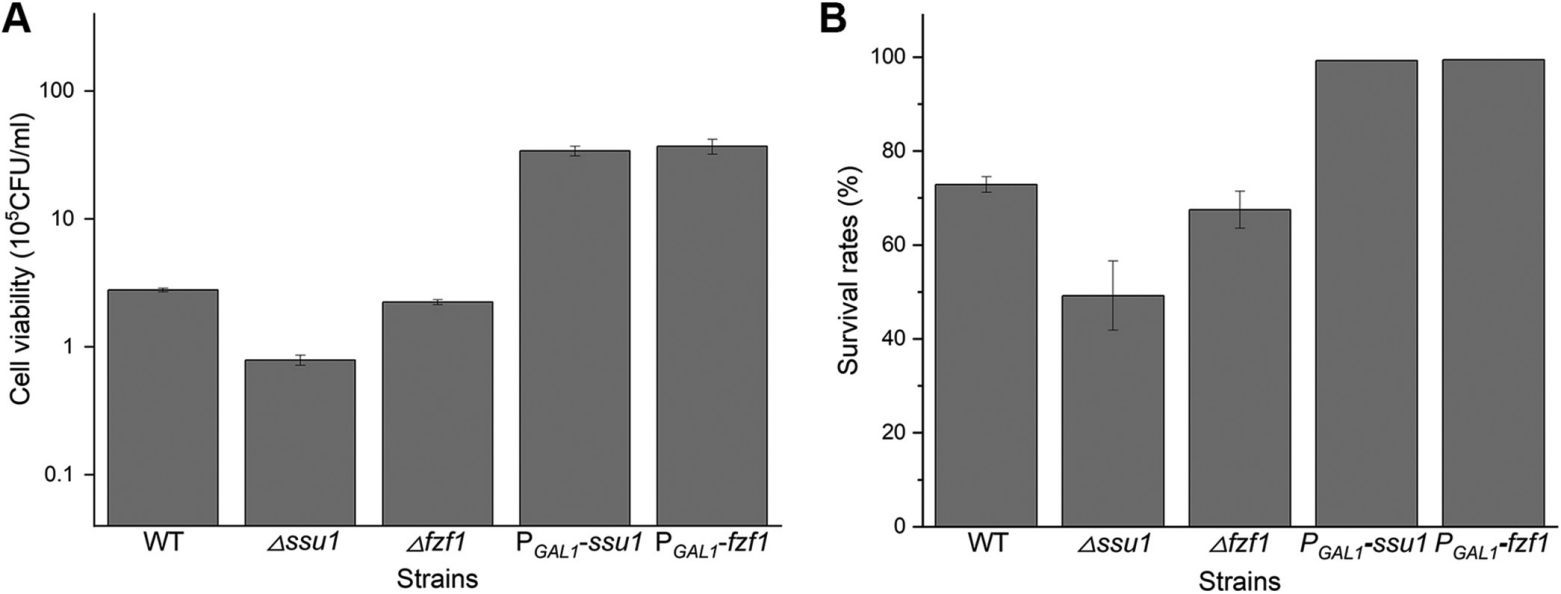
*ssu1* and *fzf1* contribute to selenium adaptation in S. cerevisiae. (A) Viability of deletion and overexpression strains after selenium treatment. Cell samples from all tested strains were harvested after culture in YPG medium with 100 μM Na_2_SeO_3_ for 16 h, diluted, and plated. After incubation at 30°C for 3 days, the number of colonies on the plates was counted. (B) Survival rates of deletion and overexpression strains after selenium treatment. Yeast cells were sampled during serial transfer in selenium-containing YPG medium, stained with propidium iodide, and analyzed by flow cytometry. The proportion of unstained cells was regarded as alive. The error bars represent the means and ranges from three independent experiments.

To verify their contributions to selenium adaptation and tolerance, reverse-engineered deletion and overexpression strains of *ssu1* and *fzf1* were used here. For the deleted strains, after selenium treatment, the Δ*ssu1* strain displayed low cell viabilities (approximately 30% of that of the wild type [WT]) and decreased survival rates (the survival rate of the Δ*ssu1* strain is 45%, while that of the WT is almost 70% in yeast extract-peptone-galactose [YPG] medium), but the Δ*fzf1* strain did not demonstrate a distinct recession in selenium adaptation compared to the WT, as seen for the Δ*ssu1* strain (Fig. 5). However, the overexpression of *ssu1* or *fzf1* (P*_GAL1_*-*ssu1* and P*_GAL1_*-*fzf1*) led to increases in the two indices (their cell viabilities were 12-fold higher than that of the WT, and their survival rates were approximately 100%). Therefore, the expression of *ssu1* and *fzf1* affected the selenium adaptation of S. cerevisiae.

**FIG 6:**
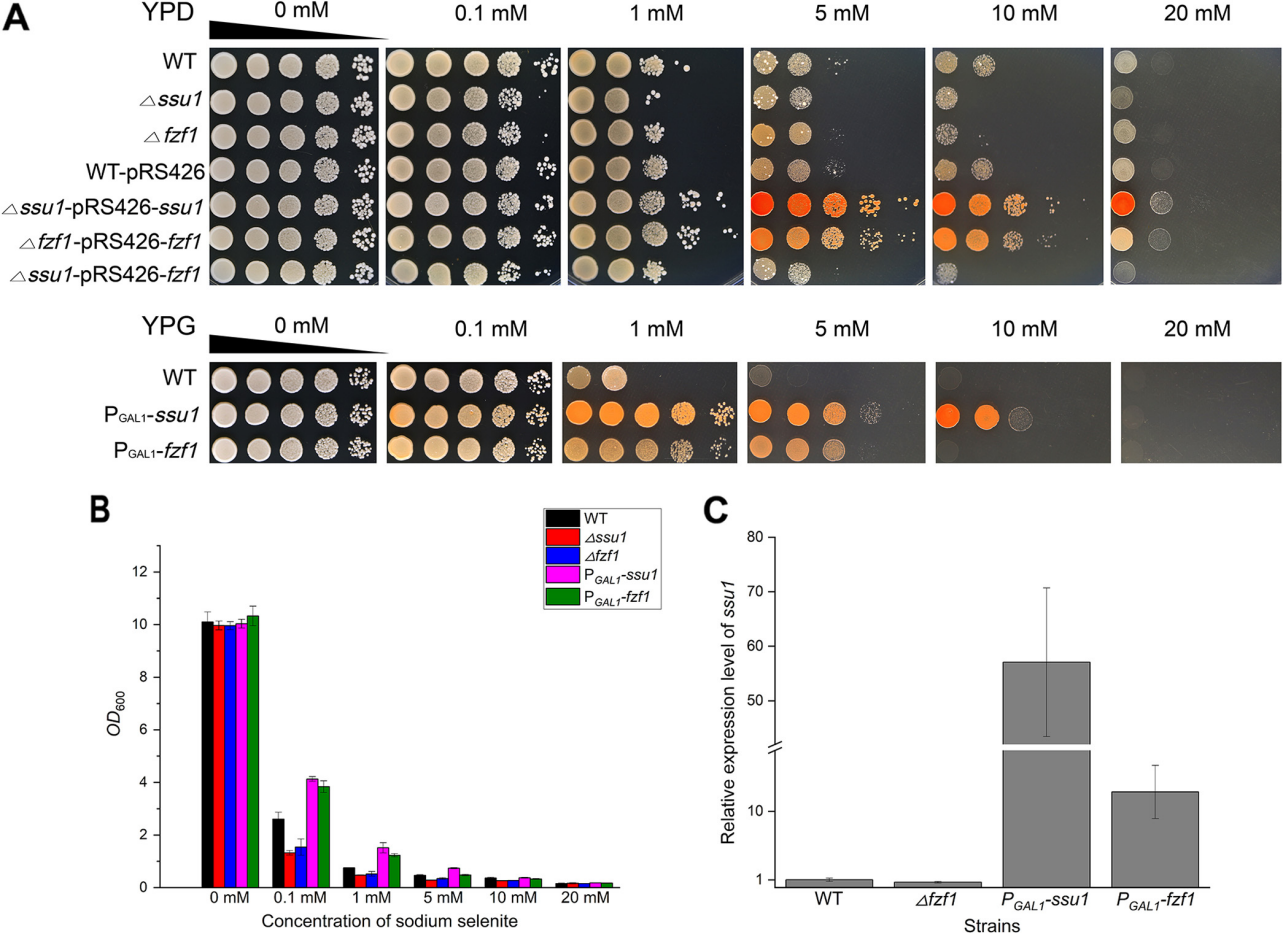
Selenium tolerance and relative expression levels of *ssu1* in the WT, Δ*fzf1*, P*_GAL1_-ssu1*, and P*_GAL1_-fzf1* strains. (A) Growth assays on agar plates. After incubation in YPD/YPG medium overnight, cells were sampled and spotted onto 1.5% YPD/YPG agar plates supplemented with increasing concentrations of Na_2_SeO_3_. Pictures were taken 2 days later. (B) Growth assays in liquid medium. All strains were grown at 30°C overnight and then subcultured into fresh YPG liquid medium with increasing concentrations of sodium selenite for 16 h. The final OD_600_ of each strain was measured and recorded. (C) The expression levels of *ssu1* in the WT, Δ*fzf1*, P*_GAL1_-ssu1*, and P*_GAL1_-fzf1* strains were determined by qRT-PCR. The error bars represent the means and ranges from three independent experiments.

Next, the effect of *ssu1* and *fzf1* on selenium tolerance was further evaluated. The deletion of *ssu1* or *fzf1* weakened the growth on selenium-containing plates, and the complemented strains (Δ*ssu1*-pRS426-*ssu1* and Δ*fzf1*-pRS426-*fzf1*) efficiently restored the selenium-tolerant phenotype, whereas the overexpression of *ssu1* or *fzf1* enhanced the growth on selenium-containing plates (Fig. 6A); the same trend was found in assays in liquid medium (Fig. 6B). However, the above-described inferior phenotypes of *fzf1* deletion and overexpression compared to *ssu1* may be because *fzf1* functions by altering the expression level of *ssu1* (Fig. 6C). The Δ*fzf1* strain caused the insufficient activation of *ssu1* and caused *ssu1* to be expressed at a lower level (approximately 0.7-fold lower than that of the WT), so its tolerance to selenium was lower than that of the WT but higher than that of the Δ*ssu1* strain. Similarly, although P*_GAL1_*-*fzf1* caused the excessive activation of *ssu1* (approximately 12.5-fold higher than that of the WT) by producing many transcription factors to bind the promoter of *ssu1*, the *GAL1* promoter in the P*_GAL1_*-*ssu1* strain is more powerful and directly upregulates the transcription of *ssu1* (approximately 57-fold higher than that of the WT). Therefore, the P*_GAL1_*-*ssu1* strain is more tolerant to selenium than the P*_GAL1_*-*fzf1* strain because of the higher expression level of *ssu1*. The above-mentioned results combined with the abortive tolerance recovery by introducing *fzf1* into the Δ*ssu1* strain (Δ*ssu1*-pRS426-*fzf1*) (Fig. 6A) comprehensively indicated that *ssu1* is the direct executant in selenium tolerance.

Moreover, the selenium-tolerant phenotype of the overexpression strains (P*_GAL1_*-*ssu1* and P*_GAL1_*-*fzf1*) is closely associated with E1 to E6, implying that the excellent selenium tolerance of the evolved strains is likely attributed to the overexpression of *ssu1* or the increased protein activity of Ssu1p. Hence, we speculated that the mutations of *ssu1* and *fzf1* screened by sequencing may be the key factors in the selenium tolerance of the evolved strains, and we demonstrated this assumption in the work described below by measuring their contributions to selenium tolerance.

### *ssu1* decreases intracellular selenium accumulation and is involved in selenium efflux.

**FIG 7:**
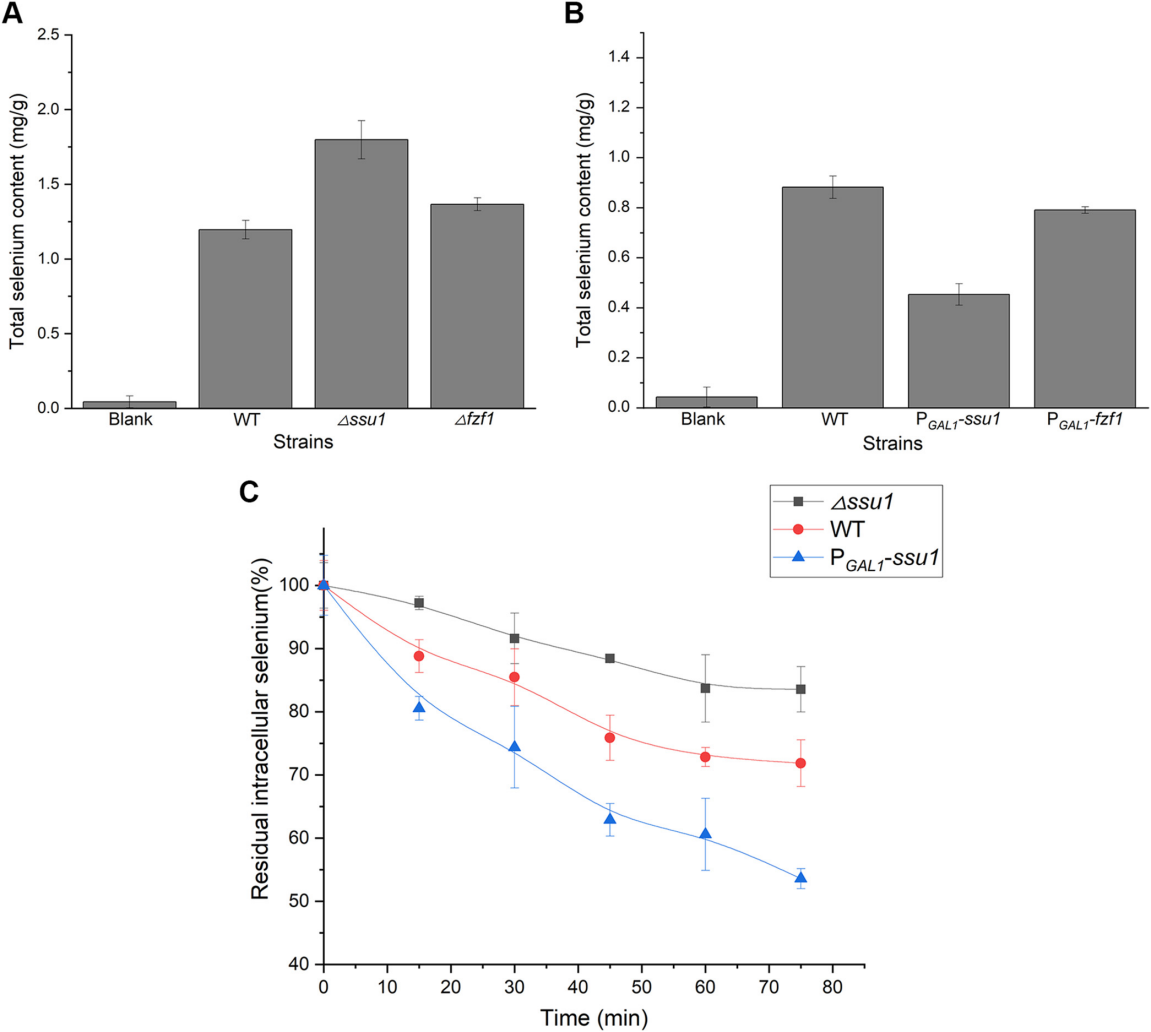
*ssu1* and *fzf1* affect intracellular selenium accumulation, and *ssu1* is involved in selenium efflux. After growth in YPD/YPG medium with 100 μM Na_2_SeO_3_, yeast cells were sampled and digested, and the selenium content was then analyzed using ICP-MS. (A) The absence of *ssu1* and *fzf1* contributes to selenium accumulation in yeast cells. (B) The overexpression of *ssu1* and *fzf1* leads to a decrease in intracellular selenium. (C) Efflux of selenium from the WT, Δ*ssu1*, and P*_GAL1_-ssu1* strains. Cells were loaded into YPG medium supplemented with 1 mM Na_2_SeO_3_ for 1 h to yield a final total intracellular concentration of 2 to 3 mg/g (dry weight). Selenium efflux was determined in Se-enriched cells suspended in fresh YPG medium by measuring the percent reduction of intracellular selenium. The error bars represent the means and ranges from three independent experiments.

To verify the specific transport function of *ssu1* in selenium tolerance, we measured the intracellular selenium contents of the Δ*ssu1*, Δ*fzf1*, P*_GAL1_*-*ssu1*, and P*_GAL1_*-*fzf1* strains. The intracellular selenium content of the Δ*ssu1* strain was 1.5-fold that of WT, while for P*_GAL1_*-*ssu1*, the selenium content was almost half that of the WT (Fig. 7A and B). Meanwhile, the effect of *fzf1* on the intracellular selenium content was the same as that of *ssu1* but was less obvious. The efflux kinetics of the WT, Δ*ssu1*, and P*_GAL1_*-*ssu1* strains were separately measured, and we found that *ssu1* affected intracellular selenium accumulation by altering the selenium efflux capabilities of yeasts. At the end of the selenium efflux measurements, the WT retained approximately 75% residual intracellular selenium, and the Δ*ssu1* strain showed a defective efflux pattern, with approximately 85% residual intracellular selenium remaining, while the P*_GAL1_*-*ssu1* strain displayed an extremely strong efflux capacity, with only 55% residual intracellular selenium remaining (Fig. 7C).

The content of intracellular selenium directly determines the toxicity induced by selenium (30); therefore, the changes in the selenium sensitivity of the Δ*ssu1* and P*_GAL1_*-*ssu1* strains may originate from the altered ability for intracellular selenium accumulation. When *ssu1* was absent (Δ*ssu1*) or downregulated (Δ*fzf1*), cells were unable to efficiently pump out toxic selenite to avoid induced damage or stress. When *ssu1* was overexpressed (P*_GAL1_*-*ssu1* and P*_GAL1_*-*fzf1*), the intracellular selenium content was maintained at a minimum level so that cells could tolerate extremely high concentrations of selenium. Our results show that the expression of *ssu1* intensely shifted the selenium efflux pattern in yeast and controlled the residual intracellular selenium content, similar to its function in sulfite metabolism (31). Consequently, we concluded that the sulfite efflux pump Ssu1p is also involved in selenium efflux and exerts a similar function.

### Mutations in Ssu1p and Fzf1p contribute to selenium tolerance in yeast.

**FIG 8:**
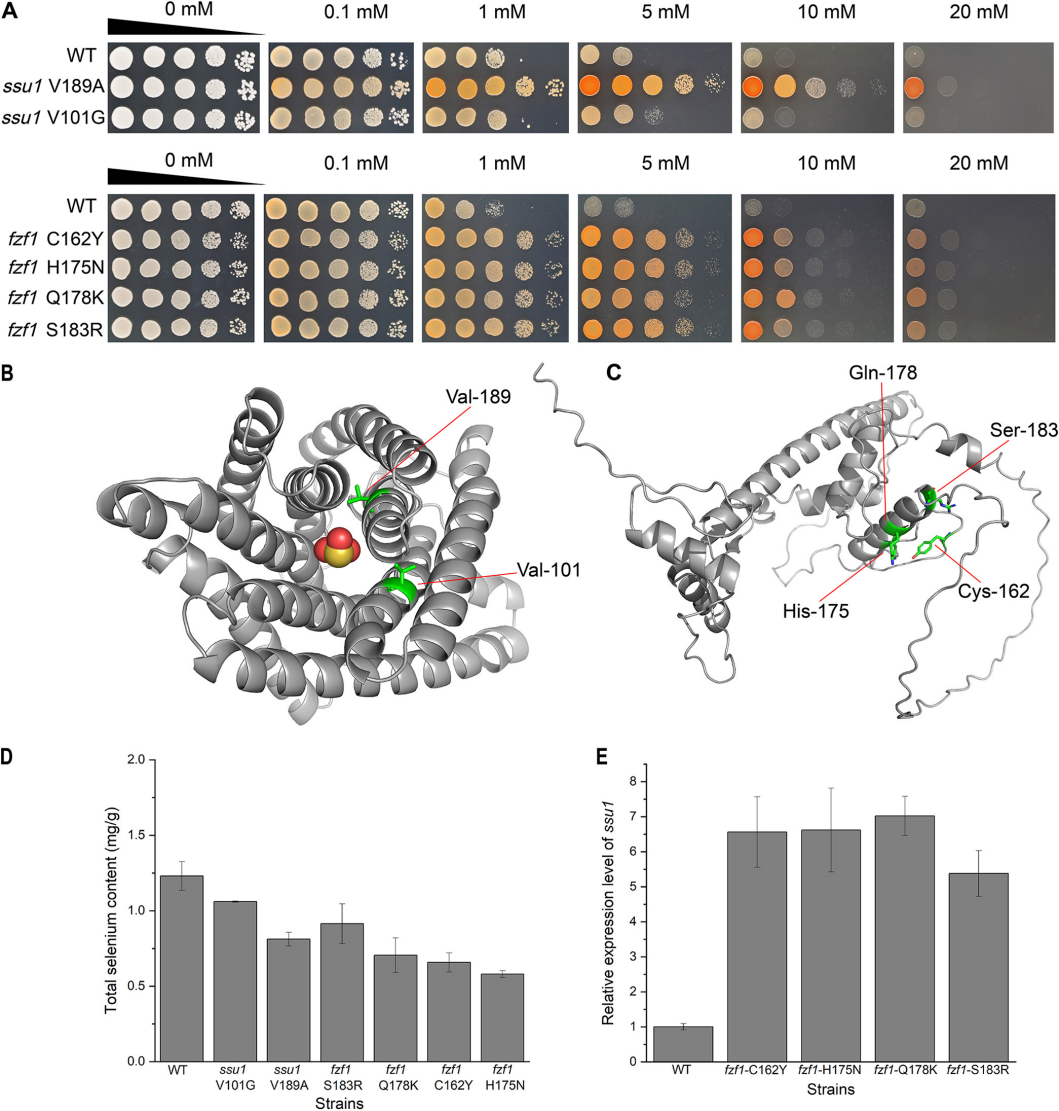
Selenium tolerance of point mutation strains and structures of Ssu1p and Fzf1p. (A) Growth assays on agar plates. After incubation in YPD medium overnight, cells were sampled and spotted onto 1.5% YPD agar plates supplemented with increasing concentrations of Na_2_SeO_3_. Pictures were taken 2 days later. (B) AlphaFold-predicted structure and functional mutated sites of Ssu1p (https://alphafold.ebi.ac.uk/entry/P41930). The central molecule represents SeO_3_^2−^. (C) AlphaFold-predicted structure and functional mutated sites of Fzf1p (https://alphafold.ebi.ac.uk/entry/P32805). (D) Measurement of the intracellular selenium contents of mutated strains. (E) Expression levels of *ssu1* in mutated *fzf1* strains.

After identifying the essential efflux function of *ssu1* for selenite, we began to investigate the point mutations screened from the evolved groups and tried to quantify their contributions to the hyper-selenium tolerance of yeast. Four missense mutations of *ssu1* and *fzf1* existed in E1 to E6 (Fig. 4C). We separately introduced these mutations into the WT and performed a selenium tolerance assay. Next, two mutations of *ssu1* (V101G and V189A) and all four mutations of *fzf1* (C162Y, H175N, Q178K, and S183R) were found to confer a considerable enhancement in the selenium tolerance of yeast (Fig. 8A). These mutations not only enabled yeast cells to grow well on YPD agar plates supplemented with up to 10 mM Na_2_SeO_3_ but also allowed cells to accumulate less intracellular selenium than in WT cells (Fig. 8D). The growth of the *ssu1* V189A mutant was more vigorous against selenite, with a low selenium content compared to that of *ssu1* V101G, which may indicate their different contributions to the enhancement of selenium tolerance. All point mutations of *fzf1* share similar phenotypes against selenite on plates and decreased intracellular selenium contents, and they all contribute to the expression of *ssu1* (Fig. 8E). Undoubtedly, these mutations are functional and significant for the selenium tolerance of E1 to E6.

The structures predicted by the AlphaFold Protein Structure Database are shown in Fig. 8B. Ssu1p is a transmembrane protein composed of 10 surrounding α-helices and a central transport channel, and the two mutations (V101G and V189A) were located exactly in the channel center or active site of anion transport. Considering that the two substitutions induced by mutations were all from complex and large amino acids (valine) to simple and small amino acids (glycine and alanine), we reasonably suspected that the two mutations may expand the size of the channel center by relieving the steric hindrance produced by the original valine. As shown in Table S4, after evaluating the binding energy of selenite and the channel center of Ssu1p by using AutoDock, we found that the two mutations (V101G and V189A) in Ssu1p both resulted in higher binding energies than that of the WT, indicating that they may lead to an increased affinity for selenite in mutated Ssu1p. Therefore, the efflux ability of point-mutated Ssu1p was likely enhanced so that the mutated strains could tolerate higher selenium stress.

Fzf1p is a transcription factor that contains five ZFs of the Cys_2_His_2_ type with striking similarity to the TFIIIA-like zinc finger motif (32). As described in a previous study (33), the fourth ZF of glioblastoma protein (Glip), which is also a five-ZF protein, was involved in DNA recognition. Based on the similarity to Glip, a point mutation of the fourth ZF of Fzf1p was found to detrimentally affect the DNA binding activity (34). Hence, it is likely that the fourth ZF of Fzf1p, ranging from 99 to 182 amino acids (aa) (26), may have an important function in the DNA binding process to impact the expression of *ssu1*. The four mutations of Fzf1p found in this study (C162Y, H175N, Q178K, and S183R) are all located in the fourth ZF region (Fig. 8C). Moreover, the mutations C162Y and H175N are adjacent in space and are exactly part of the fourth ZF motif (shown in Fig. S3), while the other two mutations nearby (Q178K and S183R) are involved in amino acid charge transformation, so they all may lead to the collapse of the fourth ZF motif and consequently improve the expression of *ssu1*.

Thus, we have verified that the mutations found in Ssu1p (V101G and V189A) may result in efflux activity promotion, and the mutations found in Fzf1p (C162Y, H175N, Q178K, and S183R) could presumably strengthen the expression of *ssu1* by increasing the DNA binding activity. Therefore, the hyper-selenium tolerance of E1 to E6 may come from a combined effect of mutated *ssu1* and *fzf1*.

### Selenite is a competitive substrate for sulfite in the efflux process mediated by *ssu1*.

**FIG 9:**
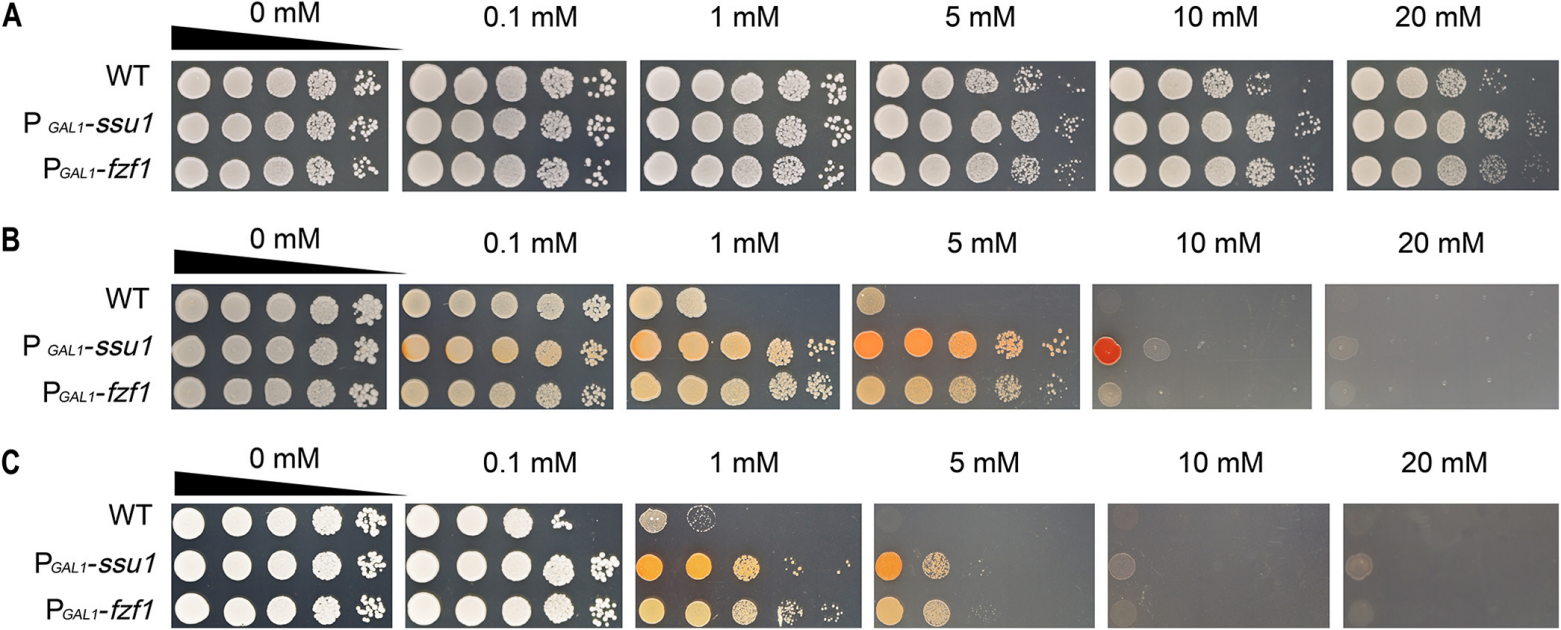
The addition of sulfite impairs the selenite tolerance of yeast on YPG agar plates. (A) The growth inhibition of all tested strains was very limited on YPG agar plates with the addition of Na_2_SO_3_. (B) On YPG agar plates with the addition of Na_2_SeO_3_, the growth of the WT was severely inhibited, while the P*_GAL1_*-*ssu1* strain could still tolerate up to 10 mM sodium selenite. (C) After incubation with 1 mM Na_2_SO_3_ for 6 h, yeast cells were washed, diluted, and spotted onto YPG agar plates with the addition of Na_2_SeO_3_, and the growth of all tested strains was inhibited further. The WT can only be sustained with 1 mM, and the P*_GAL1_*-*ssu1* and P*_GAL1_*-*fzf1* strains can only be maintained with 5 mM.

Next, the relationship between the two substrates during their efflux process was explored as our above-described results indicated that the sulfite efflux pump Ssu1p can also function in selenite transport. The growth inhibitions caused by sulfite, selenite, and both were simultaneously measured (Fig. 9). Sulfite shows a very limited inhibition effect on yeast growth; all tested strains can grow well on agar plates with 20 mM sulfite (Fig. 9A), while with the addition of selenite, the growth of the WT was severely suppressed and could be partially restored by the overexpression of *ssu1* or *fzf1* (Fig. 9B). When yeast cells were enriched with enough sulfite before being spotted onto selenium-containing YPG agar plates, all strains (including P*_GAL1_*-*ssu1* and P*_GAL1_*-*fzf1*) showed a distinct growth decline even with 1 mM selenite (Fig. 9C).

Typically, selenium metabolism always refers to the pathways of sulfur (4), and selenite reduction possibly related to upregulated sulfite metabolism pathways has also been reported (35). The sulfite level in the medium had an impact on selenite toxicity (36) and even directly affected selenite uptake or transport (37–39). In our experiment, the presence of intracellular sulfite greatly weakened the selenite tolerance of yeast. When *ssu1* was overexpressed (P*_GAL1_*-*ssu1* and P*_GAL1_*-*fzf1*), cells acquired a more powerful efflux capacity so that selenite and sulfite could be effulged promptly, and the impairment of selenite tolerance induced by sulfite could consequently be relieved. Therefore, we suggest that selenite may be a competitive substrate for sulfite during the efflux process mediated by *ssu1*, and the existence of sulfite would greatly depress the efflux efficiency of selenite and increase the selenite sensitivity of yeast.

### The expression of *ssu1* is induced in response to selenite rather than sulfite.

**FIG 10:**
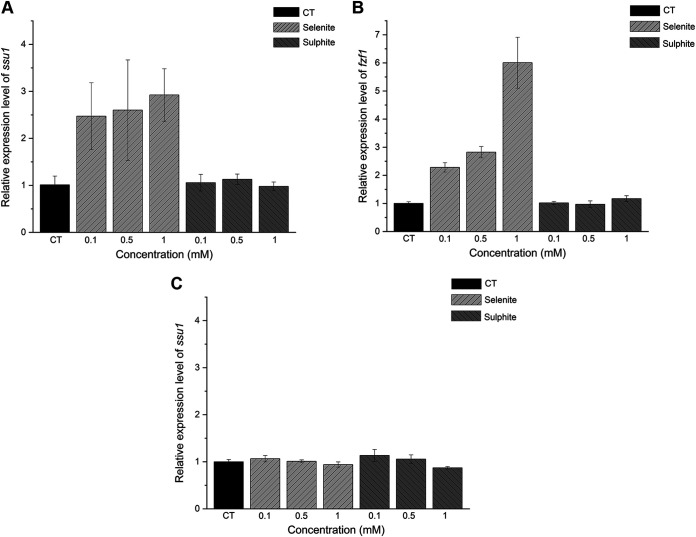
The expression level of *ssu1* responds to selenite rather than sulfite in S. cerevisiae. (A) Expression levels of *ssu1* in S. cerevisiae with different concentrations of sodium selenite or sodium sulfite. (B) Expression levels of *fzf1* in S. cerevisiae with different concentrations of sodium selenite or sodium sulfite. (C) Expression levels of *ssu1* in the Δ*fzf1* strain with different concentrations of sodium selenite or sodium sulfite. Yeast cells were incubated in YPD medium for 16 h at 30°C, different concentrations of selenite or sulfite were then added, and the cells were incubated for 2 h at 30°C. The same volume of water was added for the control groups. Finally, cells were harvested, and total RNA was extracted for qRT-PCR analysis.

Although selenite and sulfite share the same efflux pathway mediated by *ssu1*, the expression model of *ssu1* for different substrates remains to be discovered. After induction by selenite in the medium for 2 h, the expression level of *ssu1* was upregulated, and the inductive effect was gradually improved with increasing selenite concentrations (Fig. 10A). Finally, *ssu1* could be upregulated up to 2.9-fold compared to the control with 1 mM sodium selenite. In contrast, after treatment with different concentrations of sulfite, cells did not show a distinct transcription discrepancy, so sulfite seems to have little influence on the expression of *ssu1*, consistent with the results of a previous report (40). With the same pattern as that of *ssu1*, the expression level of *fzf1* was gradually upregulated by increasing concentrations of selenite (6-fold higher than that of the control in 1 mM sodium selenite) (Fig. 10B). However, when *fzf1* is absent, the expression level of *ssu1* cannot be altered, implying that the response of *ssu1* to sodium selenite is mediated by its transcription factor *fzf1* (Fig. 10C).

Although the expression of *ssu1* can lead to an increase in the sulfite tolerance of yeast (41), sulfite itself is not a powerful inducer of *ssu1* transcription (42). Notably, the expression of *ssu1* is induced in response to selenite rather than sulfite, and this response is likely regulated by its sole transcription factor, *fzf1*. The different responses of cells to selenite and sulfite may come from their divergence of toxicity as the stress imposed by selenite is more urgent and lethal than that imposed by sulfite.

### Blocking the selenium efflux process mediated by *ssu1* is a potential way to increase intracellular SeMet in Se-enriched yeast.

The efficiency of selenium enrichment is one of the most important qualities of Se-enriched yeast (43). The selenium efflux process mediated by Ssu1p would have negative effects on the production of Se-enriched yeast, which will result in the severely low utilization of raw Se sources. In our work, intracellular selenium accumulation was found to be closely related to the expression of *ssu1*, and the deletion of *ssu1* led to an increased content of intracellular selenium, implying a feasible way to advance Se-enriched yeast production by blocking selenium efflux. Hence, based on the deletion of *ssu1*, we optimized yeast strains to gain more advantageous Se-enriched characteristics, such as the most stable and valuable intracellular organic form of selenium—SeMet.

**FIG 11:**
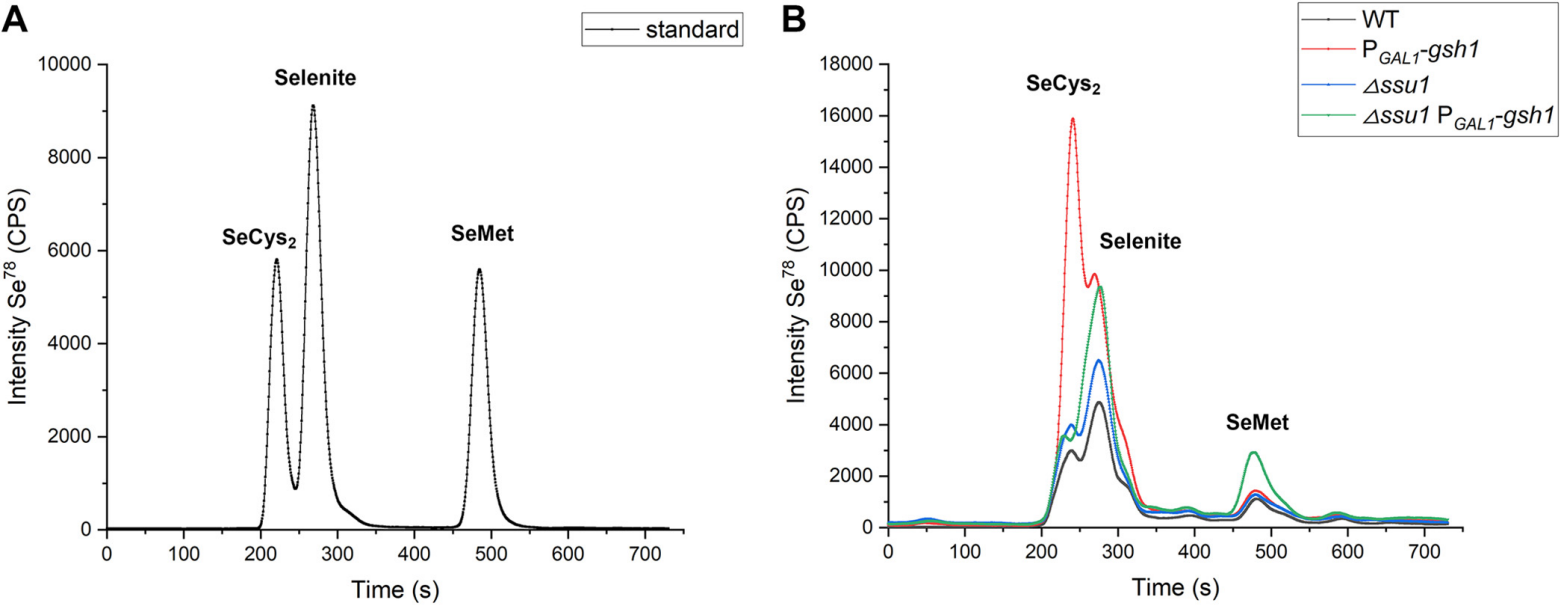
Characterization of selenium species of Se-enriched yeasts by reversed-phase HPLC–ICP-MS. (A) Chromatograms of selenium species standards (150 ppb) of SeMet (selenomethionine), SeCys_2_ (selenocystine), and selenite. (B) Chromatograms of the tested yeast strains. All tested strains were inoculated into YPG medium containing 0.1 mM sodium selenite and cultivated for 16 h before being sampled and analyzed.

To ensure abundant reducing power in yeast cells to convert inorganic selenium into organic selenium, we overexpressed *gsh1* (encoding γ-glutamylcysteine synthetase, which catalyzes the first step in glutathione biosynthesis) in the Δ*ssu1* strain. Compared to the WT, both the Δ*ssu1* and P*_GAL1_*-*gsh1* strains had higher intracellular levels of SeCys_2_ and more unconverted inorganic selenium, but they seemed not to increase the SeMet content (Fig. 11B). However, when they were combined, our modified Δ*ssu1*-P*_GAL1_*-*gsh1* strain successfully achieved increased SeMet yields, approximately twice those of the other three strains, meaning that intracellular trapped inorganic selenium can be efficiently transformed into SeMet by enough reduced glutathione. Therefore, our strain construction strategy works well and demonstrates the considerable potential for the industrial production of Se-enriched yeast, especially for intracellular SeMet.

### Conclusion.

In this study, through the adaptive laboratory evolution of sodium selenite, we obtained selenium-tolerant strains and demonstrated that the combined effect generated by mutated Ssu1p and Fzf1p was sufficient to confer selenium tolerance to S. cerevisiae. For the first time, the selenium efflux process mediated by *ssu1* has been described. Ssu1p helps yeast cells protect against selenium by decreasing intracellular selenium accumulation, while its transcription factor Fzf1p exerts the same function by adjusting the expression level of *ssu1*. We found that selenite is a competitive substrate for sulfite during the efflux process, while the expression of *ssu1* responds to selenite rather than sulfite. Moreover, based on the deletion of *ssu1* and additional intracellular reducing power, we achieved higher intracellular SeMet concentrations in yeast, which may contribute to the industrial production of Se-enriched yeast.

In conclusion, our results suggest a latent selenium transport pathway through the efflux process, which would be a significant complement to the understanding of selenium metabolism and application.

## MATERIALS AND METHODS

### Yeast strains and media.

The S. cerevisiae strains used in this study are derived from BY4742 (*MAT***a** *his3*Δ*1 leu2*Δ*0 ura3*Δ*0*), obtained from Euroscarf. All yeast strains used in this work are listed in Table 1. YPD medium contained 1% yeast extract (BD, USA), 2% peptone (BD, USA), and 2% dextrose (Amresco, USA). YPG medium contained 1% yeast extract (BD, USA), 2% peptone (BD, USA), and 2% galactose (Amresco, USA). All cultivations were carried out at 30°C.

**TABLE 1:** List of S. cerevisiae strains used in this work

Strain	Relevant genotype	Source or reference
WT	BY4742 (*MAT***a** *his3*Δ*1 leu2*Δ*0 ura3*Δ*0*)	Euroscarf
Δ*ssu1*	BY4742 *ssu1*Δ::*KANMX6*	This work
Δ*fzf1*	BY4742 *fzf1*Δ::*KANMX6*	This work
WT-pRS426	BY4742/pRS426	This work
Δ*ssu1*-pRS426*-ssu1*	BY4742 *ssu1*Δ::*KANMX6*/pRS426-*ssu1*	This work
Δ*fzf1*-pRS426*-fzf1*	BY4742 *fzf1*Δ::*KANMX6*/pRS426-*fzf1*	This work
Δ*ssu1*-pRS426*-fzf1*	BY4742 *ssu1*Δ::*KANMX6*/pRS426-*fzf1*	This work
Δ*ssu1*-P*_GAL1_-gsh1*	BY4742 *ssu1*Δ::*KANMX6* P*_GAL1_-gsh1*::*URA3*	This work
P*_GAL1_-ssu1*	BY4742 P*_GAL1_-ssu1*::*KANMX6*	This work
P*_GAL1_-fzf1*	BY4742 P*_GAL1_-fzf1*::*KANMX6*	This work
P*_GAL1_-gsh1*	BY4742 P*_GAL1_-gsh1*::*URA3*	This work
*ssu1* V189A	BY4742 *ssu1*-V189A::*URA3*	This work
*ssu1* V101G	BY4742 *ssu1*-V101G::*URA3*	This work
*ssu1* I127T	BY4742 *ssu1*-I127T::*URA3*	This work
*ssu1* L259F	BY4742 *ssu1*-L259F::*URA3*	This work
*fzf1* C162Y	BY4742 *fzf1*-C162Y::*URA3*	This work
*fzf1* H175N	BY4742 *fzf1*-H175N::*URA3*	This work
*fzf1* Q178K	BY4742 *fzf1*-Q178K::*URA3*	This work
*fzf1* S183R	BY4742 *fzf1*-S183R::*URA3*	This work

### Adaptive laboratory evolution.

Selenium-tolerant strains were generated from strain BY4742 (*MAT***a** *his3*Δ*1 leu2*Δ*0 ura3*Δ*0*) by serial cultivations in YPD medium containing increasing amounts of sodium selenite for 100 days (Fig. 1). The design of laboratory evolution refers to methods in a previous study (10). Specifically, the parental strain was chosen from a single colony of BY4742 and inoculated into YPD medium with agitation at 200 rpm overnight at 30°C. The culture grown overnight was denoted S (starting strain), and the cells were then reinoculated into nine 250-mL flasks containing 50 mL fresh YPD with agitation at 200 rpm overnight at 30°C. The nine cultures were serially propagated in YPD medium after 24 h independently (adjusting the initial optical density at 600 nm [OD_600_] to 0.2). Six cultures were supplemented with 50 μM sodium selenite and denoted Ev1 to Ev6 (evolved group) to endow yeast with selenite resistance, while the other three cultures were not and were denoted CT1 to CT3 (control group) to eliminate interference from other factors. When adaptive evolution was conducted for 50 days, another 50-day serial passage was performed to further enhance the selenium resistance phenotype of the evolved groups, and the selenium concentration was increased to 100 μM. In addition, to prevent occasional contamination, the cultures were periodically sampled for microscopic observation and stored in 25% glycerol at −80°C. Finally, the evolution of Ev1 to Ev6 was conducted under continuous sodium selenium for 100 days, and six strains from the final evolved groups were isolated for the following research and denoted E1, E2, E3, E4, E5, and E6.

### Strain construction.

The primers and plasmids used in this study are listed in Tables S1 and S3 in the supplemental material, respectively. The deletion strains were constructed by replacing the entire reading frame with a PCR-generated G418 resistance marker cassette (*KANMX*), and the complemented strains were constructed by introducing yeast expression plasmid pRS426 containing the corresponding gene into the deletion strains. The yeast shuttling expression plasmid pRS426 has an *AmpR* marker for selection in Escherichia coli and a *URA3* marker for selection in yeast, and we placed a *TEF* promoter and a *CYC1* terminator at the multiple-cloning site to control the expression of the inserted gene. The overexpression strains were constructed by integrating a *URA3*-targeted *GAL1* promoter in front of the gene. For the point mutant strains, site-directed PCR mutagenesis was performed to replace the position of interest coupled with a *URA3* marker. The manipulation of plasmid DNA or genomic DNA and the transformation of yeast cells were performed according to standard procedures (44).

### Selenite tolerance assay.

For the growth assays on agar plates, the tested strains were inoculated into YPD liquid medium and cultivated for 16 h (during the exponential growth phase). Next, all cultures were adjusted to an OD_600_ of 6.0 and washed twice with sterile water. After serial dilution with sterile water, 3 μL of each dilution was spotted onto 1.5% YPD or YPG agar plates supplemented with various concentrations of sodium selenite. Pictures were taken after 2 days of incubation at 30°C.

For the growth assays in liquid medium, the tested strains were grown at 30°C overnight and then subcultured into fresh YPD or YPG liquid medium with various concentrations of sodium selenite. After 16 h of incubation, the final OD_600_ of each strain was measured and recorded.

### Cell viability and survival tests.

To estimate the cell viability of each strain after selenite treatment, cultures of growing yeast cells were sampled after 16 h of incubation in selenium-containing liquid medium, adjusted to an OD_600_ of 6.0, and washed twice with sterile water. After proper dilution, cultures of each strain were plated onto 1.5% YPD agar plates. The plates were incubated at 30°C for 3 days, and the number of colonies was counted.

The cell survival rates of each strain after selenite treatment were measured by flow cytometry. After 16 h of incubation in selenium-containing liquid medium, 6 × 10^7^ cells were harvested by centrifugation, washed three times with deionized water, resuspended in 1 mL of 0.85% (wt/vol) NaCl, and stained with 20 μL of the fluorescent probe propidium iodide (PI) (100 μg/mL) for 15 min. Stained cells were analyzed by using a flow cytometer (Beckman Cytoflex), and the fluorescence emission was measured in the logarithmic mode for the PI signal. Unstained and ethanol-treated cells were used as controls. The flow rate during analysis never exceeded 1,000 cells s^−1^. A total of 2 × 10^4^ cells were measured for each sample. The proportion of PI-stained cells was regarded as dead cells, while the other proportion was counted as alive.

### Whole-genome sequencing and analysis.

Genomic DNA samples of six single colonies from the evolved strains (E1, E2, E3, E4, E5, and E6) and one clone from the starting strain (S) were isolated using the Tianamp yeast DNA kit (Tiangen, China) according to the manufacturer’s recommendations.

The DNA samples were subjected to whole-genome resequencing using the Illumina NovaSeq platform with paired-end 2× 150-bp reads targeting a genome coverage of 130× per sample. The processed reads were mapped to the S. cerevisiae BY4742 reference genome (GenBank accession number JRIR00000000.1) with the aligner BWA (version 0.7.12). To improve the accuracy of the prediction of single nucleotide polymorphisms (SNPs), PICARD software (version 1.107) and the GATK package were applied to remove potential errors in sequencing and mapping. SNPs and indels (insertions-deletions) were detected by using GATK and annotated by using ANNOVAR, while the CNV (copy number variation) and SV (structural variation) were investigated using CNVnator (version 0.2.7) and Break Dancer (version 1.1), respectively. Finally, the total variations between S and E1 to E6 were counted and analyzed, and the potential mutated site was determined for further functional verification.

### Selenium efflux and intracellular selenium content quantification.

After selenium treatment, 600 μL of yeast cells was rapidly harvested by centrifugation and washed with ice-cold MilliQ water at least three times. Next, the samples were dried in a metal bath at 80°C to obtain the dry weight. To determine selenium efflux, cells during the exponential growth phase were incubated in YPG medium supplemented with 1 mM sodium selenium at 30°C for 1 h, and the cells were then rapidly filtered and washed with ice-cold 75 mM tartaric acid (pH 3.5). Selenium efflux was initiated by resuspending the cell pellet in fresh YPG medium with agitation at 30°C. At appropriate time intervals, 1-mL aliquots were taken and rapidly collected for further measurements.

The samples were digested with 1 mL 65% nitric acid at 120°C for 2 h, diluted properly, and measured by inductively coupled plasma mass spectrometry (ICP-MS). The results are the means of data obtained for each isotope and are expressed in milligrams per gram (selenium content/dry weight).

### qRT-PCR analysis.

RNA extraction was conducted according to a method described in a previous study (45). The CFX384 real-time PCR detection system (Bio-Rad Laboratories, CA) was used to perform quantitative reverse transcription–real-time PCR (qRT-PCR) experiments with SYBR quantitative PCR (qPCR) master mix (Vazyme, China). The thermocycler program used was 95°C for 3 min followed by 39 cycles of 95°C for 30 s and 60°C for 1 min. *ACT1* was chosen as the reference gene, and the primers used in the qRT-PCR experiments are listed in Table S2. Three parallel experiments were conducted for each gene, and the 2^−ΔΔ^*^CT^* method was employed to analyze the data.

### Measurement of selenium species.

To determine the intracellular organic selenium contents, especially SeCys_2_ and SeMet, 6 mL of yeast cells was harvested by centrifugation, washed, and resuspended in 1× phosphate-buffered saline (PBS) (pH 7.5). Cells were disrupted using a Mini-Bead beater-16 instrument for 30 s and cooled on ice for another 30 s, four times. After centrifugation, the supernatant of the lysates was collected, trypsin (1-mg/mL final concentration) and TCS buffer (10×) (0.1 mM Tris-HCl, 10 mM CaCl_2_, 0.5% SDS) were added, and the samples were incubated at 37°C for 20 h. Next, the samples were further treated with pronase E (1-mg/mL final concentration) for 20 h and finally filtered with 0.22-μm filters to remove undigested proteins. Reversed-phase high-performance liquid chromatography (HPLC)–ICP-MS measurements were performed using an ICP-MS system (Thermo Scientific, USA) for online element-specific detection, coupled with an HPLC system (Dionex, USA) equipped with a Hypersil BDS C_18_ column (3 μM, 150 mm by 2.1 mm; Thermo Scientific, USA).

### Data availability.

All of the data and materials generated in this study can be provided upon a request submitted to the corresponding author for at least 5 years following the publication date.
