# Supplementary material for: Adaptive Laboratory Evolution Reveals the Selenium Efflux Process To Improve Selenium Tolerance Mediated by the Membrane Sulfite Pump in Saccharomyces cerevisiae
Source: Microbiol Spectr. 2023 Apr 26;11(3):e01326-23. doi: 10.1128/spectrum.01326-23 (PMC10269739; doi:10.1128/spectrum.01326-23)
Supplement: Supplemental file 1 — Supplemental material. Download spectrum.01326-23-s0001.docx, DOCX file, 6.8 MB [file spectrum.01326-23-s0001.docx]

Supplementary data

**Adaptive laboratory evolution reveals the selenium efflux process to improve selenium tolerance mediated by membrane sulfite pump in *Saccharomyces cerevisiae***

Ao Gong, ^a,b^ Wenyue Liu, ^a,b^ Yelong Lin, ^a,b^ Laili Huang, ^a,b^ Zhixiong Xie^a,b^**^*^**

^a^ Hubei Key Laboratory of Cell Homeostasis, Wuhan University, Wuhan, China

^b^ College of Life Sciences, Wuhan University, Wuhan, China

*E-mail address*: [zxxie@whu.edu.cn](mailto:zxxie@whu.edu.cn) (**^*^**Correspondence, Z.X.)

**
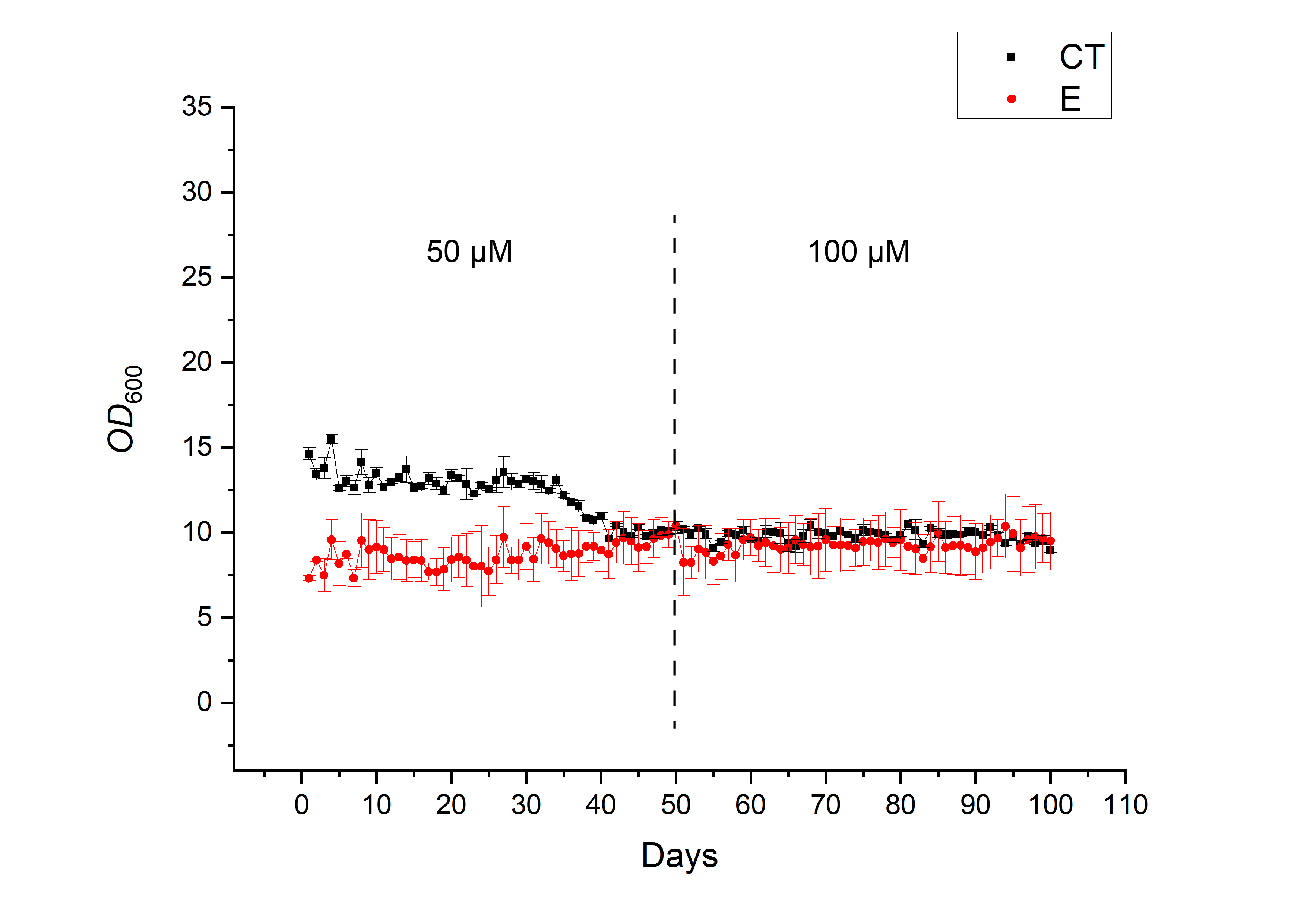
**

**Fig. S1.** The daily growth monitoring of control groups (CT) and evolved groups (E). The selective pressure for the first 50 days was 50 μM of Na_2_SeO_3_, and for the last 50 days was 100 μM. The evolved groups gradually exhibited growth enhancement under the selective pressure of Na_2_SeO_3_, while the control groups degenerated in nutritious YPD medium without selective pressure.


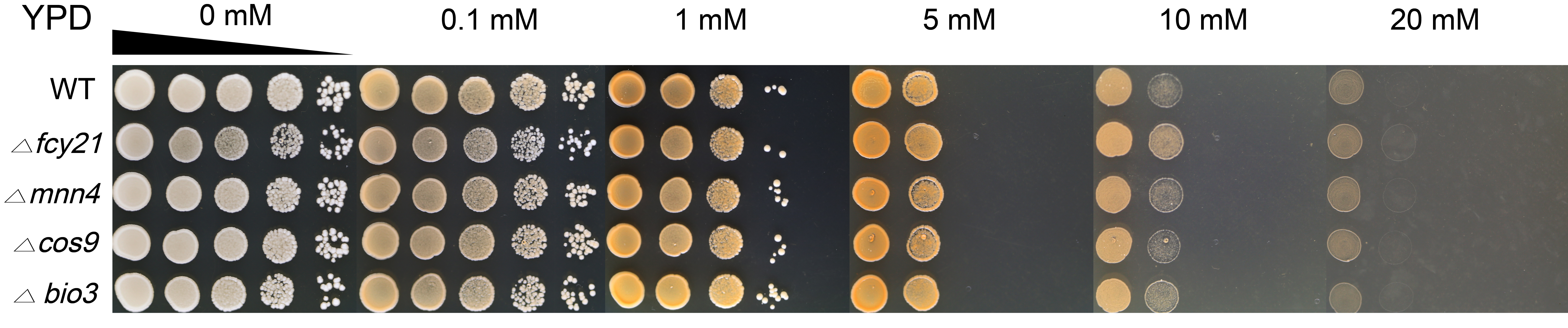


**Fig. S2.** Growth assays on agar plates of the deletion strains (*Δfcy21*, *Δcos9*, *Δmnn4, Δbio3*) and these genes were found to be irrelevant to selenium tolerance. After overnight incubation in YPD medium, cells were sampled and spotted on 1.5% YPD agar plates supplemented with an increasing concentration of Na_2_SeO_3_. Pictures were taken 2 days later.


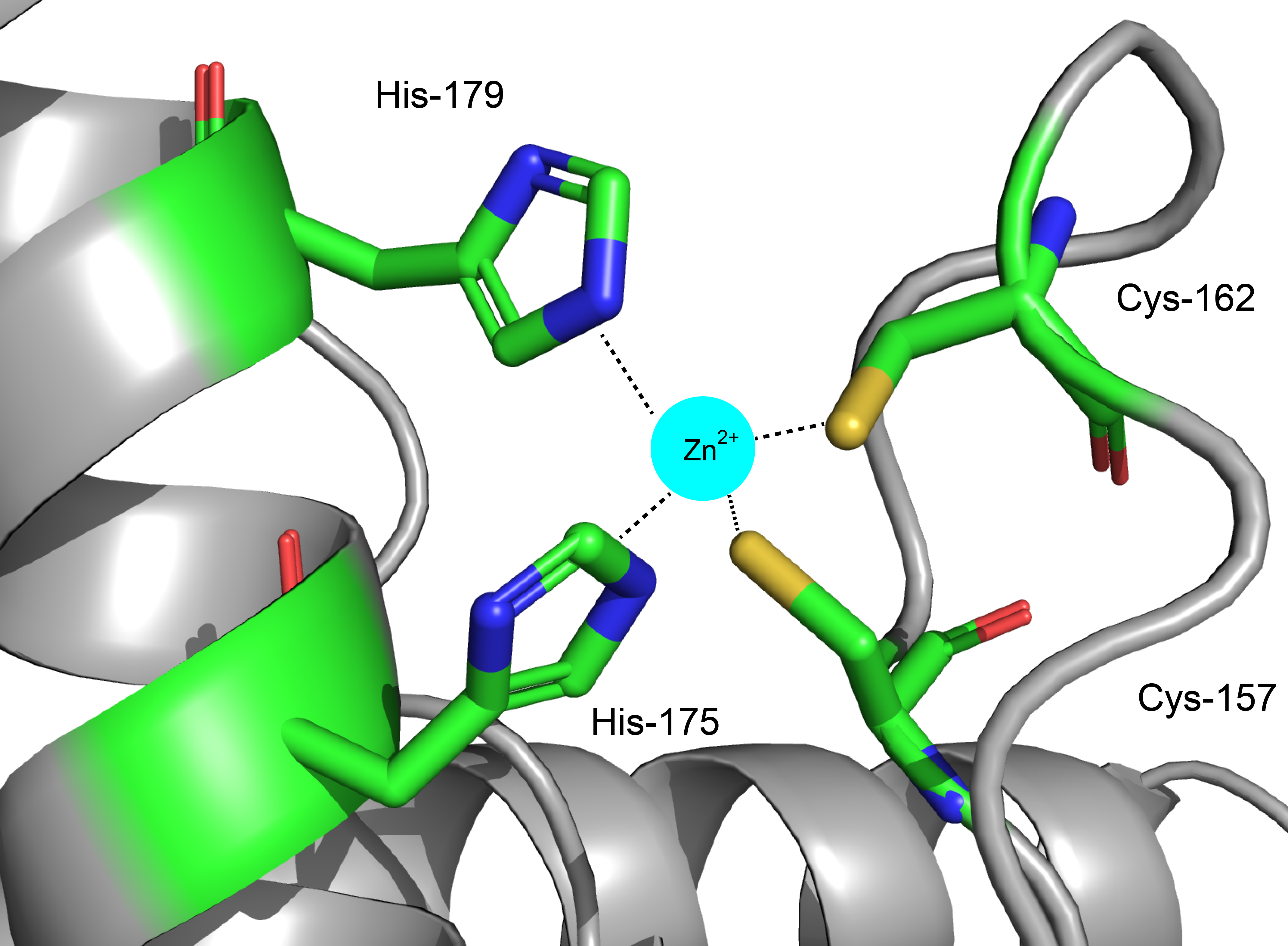


**Fig. S3.** The fourth zine finger motif contained Cys-162 and His-175 in Fzf1p.

**Table S1**

List of primers used in this work.

| Primer name | Primer sequence (5’-3’) | Description |
| --- | --- | --- |
| SSU1-DEL-UP | GCAATTTAAAAACGTTTTATAGTGTAAGAGAAGACAAGTACAAGAAAAAAATACAACGTATGCAATGGGC | Deletion of *ssu1* |
| SSU1-DEL-DOWN | GACTCCTACATGAAATGCTTGCCAATTATGTACGTATAAAACCAACAATTGGCCACTAGTGGATCTGATA |  |
| SSU1-CKA | AAACTTGTGATATTGGCTGAAC | PCR verifies deletion of *ssu1* |
| SSU1-CKB | CCCCACGCTATTACTAGACAGAC |  |
| SSU1-CKC | CTGCTATGGCATTGCTCG |  |
| SSU1-CKD | ATTGACAAGGGTCGTGCT |  |
| SSU1-PGAL-UP | GATTGAGCTCAGACAATACGCGCAATTTAAAAACGTTTTATAGTGTAAGAGAATTCGAGCTCGTTTAAAC | Overexpression of *ssu1* |
| SSU1-PGAL-DOWN | AACATGAAGGGGTCAAACTGCCTCGTAAGAGCAAGTACCCAATTGGCAACCATTTTGAGATCCGGGTTTT |  |
| FZF1-CKA | CTTTGATCGGTAAGTGGGC | PCR verifies deletion of *fzf1* |
| FZF1-CKB | TTCGCATTCACTCCTTCGT |  |
| FZF1-CKC | AAGTGCTACAATGCCCATAC |  |
| FZF1-CKD | CAATGTGCGAGGAGGTTCA |  |
| FZF1-PGAL-UP | AAAGAAGATGAAAATGACAACTTTTACGCTGGTGTGCACAAGTGGTACCAGAATTCGAGCTCGTTTAAAC | Overexpression of *fzf1* |
| FZF1-PGAL-DOWN | CCGTCAAAAGAACATTTGTAATTTCTAGACTTGGTTCTCCCTATATCCGTCATTTTGAGATCCGGGTTTT |  |
| MULTIS-SSU1-CKA | CAGGTCGACTCTAGAGGATCCAAACTTGTGATATTGGCTGAAC | Site-directed mutagenesis of *ssu1* |
| SSU1+URA-LAP-UP | TCTGATATCATCGATGAATTCTTAGTTTTGCTGGCCGCAT |  |
| SSU1+URA-LAP-DOWN | GAAAAGCTAGTTATGCTAAACGCGTAAAATCTAGAG |  |
| MULTIS-URA-DOWN | GGAATTCCTTAGTTTTGCTGGCCGCA |  |
| SSU1-V101G-UP | CCATGGGGTTAGGTACAATTAT |  |
| SSU1-V101G-DOWN | ATAATTGTACCTAACCCCATGG |  |
| SSU1-I127T-UP | AATTTGATGACATTTGTTTACGTC |  |
| SSU1-I127T-DOWN | GACGTAAACAAATGTCATCAAATT |  |
| SSU1-V189A-UP | CGCTGGTTGCCGTCGCTTC |  |
| SSU1-V189A-DOWN | GAAGCGACGGCAACCAGCG |  |
| SSU1-L259F-UP | CCTGCTTTTCGGCCCGA |  |
| SSU1-L259F-DOWN | TCGGGCCGAAAAGCAGG |  |
| SSU1-HOMO-DOWN | GACTCCTACATGAAATGCTTGCCAATTATGTACGTATAAAACCAACAATTTTAGTTTTGCTGGCCGCAT |  |
| MULTIS-FZF1-CKA | CAGGTCGACTCTAGAGGATCCCTTTGATCGGTAAGTGGGC | Site-directed mutagenesis of *fzf1* |
| FZF1+URA-LAP-UP | TTATTCGAATACTGACTAGCTTTTCAATTCAATTCATCATT |  |
| FZF1+URA-LAP-DOWN | GCTAGTCAGTATTCGAATAAATCCCAGA |  |
| MULTIS-URA-DOWN | GGAATTCCTTAGTTTTGCTGGCCGCA |  |
| FZF1-C162Y-UP | TCATATCAAAAAGTTACCAGCTT |  |
| FZF1-C162Y-DOWN | AAGCTGGTAACTTTTTGATATGA |  |
| FZF1-H175N-UP | GACGATCTAATAAATAATATGTTGCA |  |
| FZF1-H175N-DOWN | TGCAACATATTATTTATTAGATCGTC |  |
| FZF1-Q178K-UP | TGTTGAAACATCATATAGCAAGTAA |  |
| FZF1-Q178K-DOWN | TTACTTGCTATATGATGTTTCAACA |  |
| FZF1-S183R-UP | AGCAAGGAAGCTTGTTGTACC |  |
| FZF1-S183R-DOWN | GGTACAACAAGCTTCCTTGCT |  |
| FZF1-HOMO-DOWN | CATAGTTCGAATCACATGAGTAGAGGACGGAAATTGCTCTTCTATGGCGTTTAGTTTTGCTGGCCGCAT |  |
| PSP72-UP | CGGAGACGGTCACAGCT | PCR verifies the cloning of site-directed mutagenesis fragments |
| PSP72-DOWN | AGGGTCGGAACAGGAGA |  |

**Table S2**

List of qRT-PCR primers used in this work.

| Primer name | Primer sequence (5’-3’) | Description |
| --- | --- | --- |
| q-ACT1-F | TCTGCCGGTATTGACCAAAC | Analysis of gene *act1* |
| q-ACT1-R | ATGGAAGATGGAGCCAAAGC |  |
| q-SSU1-F | TTGGTCAATTCTATGCCTTTTATGC | Analysis of gene *ssu1* |
| q-SSU1-R  q-FZF1-F  q-FZF1-R | CTTCCACGCTTTCAATGCTGTTA  ACCGTGCCATCTACGAGTTCA TCCTTGCCATTGAGTTCTGCT | Analysis of gene *fzf1* |

**Table S3**

List of plasmids used in this work.

| Plasmids | Features | Source |
| --- | --- | --- |
| pUG6 | *KANMX6* | This work |
| pFA6a-kanMX6-P_GAL1_ | *KANMX6,* P*_GAL1_* | This work |
| pRS426  pRS426-*ssu1*  pRS426-*fzf1*  pSP72 | *URA3, P_TEF_ –* MCS -*T_CYC1_*  *URA3, P_TEF_ –ssu1*-*T_CYC1_*  *URA3, P_TEF_ –fzf1*-*T_CYC1_*  *AmpR* | This work  This work  This work  This work |
| pSP72-ssu1 V189A | *AmpR, ssu1*-V189A:*URA3* | This work |
| pSP72-ssu1 V101G | *AmpR, ssu1*-V101G:*URA3* | This work |
| pSP72-ssu1 I127T | *AmpR, ssu1*-I127T:*URA3* | This work |
| pSP72-ssu1 L259F | *AmpR, ssu1*-L259F:*URA3* | This work |
| pSP72-fzf1 C162Y | *AmpR, fzf1*-C162Y:*URA3* | This work |
| pSP72-fzf1 H175N | *AmpR, fzf1*-H175N:*URA3* | This work |
| pSP72-fzf1 Q178K | *AmpR, fzf1*-Q178K:*URA3* | This work |
| pSP72-fzf1 S183R | *AmpR, fzf1*-S183R:*URA3* | This work |

**Table S4**

Selenite binding energies in mutated Ssu1p were evaluated through AutoDock calculations

| Ligand | Amino acid substitution of Ssu1p | Binding energy |
| --- | --- | --- |
| Selenite (SeO_3_^2-^) | WT | -2.26 |
|  | V189A | -2.85 |
|  | V101G | -2.65 |
